# Roles of TOPO Coordinating Solvent on Prepared Nano-Flower/Star and Nano-Rods Nickel Sulphides for Solar Cells Applications

**DOI:** 10.3390/nano12193409

**Published:** 2022-09-28

**Authors:** Mojeed A. Agoro, Edson L. Meyer

**Affiliations:** 1 Fort Hare Institute of Technology, University of Fort Hare, Private Bag X1314, Alice 5700, South Africa; 2 Department of Chemistry, University of Fort Hare, Private Bag X1314, Alice 5700, South Africa

**Keywords:** molecular precursor, nickel sulphide, quantum dots, particles size, morphology

## Abstract

The present study describes a cheap, safe, and stable chemical process for the formation of nickel sulphide (NiS) with the use of mixed and single molecular precursors. The production pathway is uncomplicated, energy-efficient, quick, and toxic-free, with large-scale commercialization potential. The obtained results show the effect of tri-*N*-octylphosphine oxide (TOPO) as a coordinating solvent on the reaction chemistry, size distributions, morphology, and optical properties of both precursors. Ni[*N*,*N*-benz-*N*-*p*-anisldtc] as NiSa, Ni[*N*,*N*-benzldtc] as NiSb, and Ni[*N*-*p*-anisldtc] as NiSc thermally decompose in a single step at 333–334 °C. The X-ray diffraction peaks for NiSa, NiSb, and NiSc matched well with the cubic NiS nanoparticles and corresponded to planes of (111), (220), and (311). The extrapolated linear part from the Tauc plots reveals band gap values of 3.12 eV, 2.95 eV, and 2.5 eV, which confirms the three samples as potential materials for solar cell applications. The transmission electron microscopy (TEM) technique affirmed the quantum dot size distribution at 19.69–28.19 nm for NISa, 9.08–16.63 nm for NISb, and 9.37–10.49 nm for NISc, respectively. NiSa and NiSc show a clearly distinguishable flower/star like morphology, while NiSb displays a compact nano-rod shape. To the best of the authors’ knowledge, very few studies have been reported on the flower/star like and nano-rod shapes, but none with the dithiocarbamate molecular precursor for NiS nanoparticles.

## 1. Introduction

The current situation between Russia and most of the developed western countries, namely their sore dependence on Russian coal, fuels, and gas products, is having a serious impact on their economies. The only possible solution to these geometrical political constraints is a change to a renewable energy that is cost-effective, easy to generate, and environmentally friendly, such as solar energy. The most promising next-generation solar cells in the last two decades have been quantum dot-sensitized solar cells (QDSCs), which have reasonable efficiency and future-enhancement prospects. These future expectations are linked to their ability to optimize quantum dots (QDs) particle sizes, their cost friendliness, easy fabrication, and different wavelength ranges that will cover the visible light spectrum. Furthermore, a single photon can greatly enhance the conversion output of QDSCs through multiple-exciton excitons, making QDs a potential candidate in applications, such as solar cells [1,2,3,4,5,6,7]. Replacing the dye molecules in the dye-sensitized solar cell structure principle with QD semiconductors will increase conversion efficiency [8,9,10].

QDs semiconductors from metal chalcogenides (selenides, sulphides, tellurides) have been employed in QDSCs as photosensitizers, displaying remarkable performance, as well as in other applications such as optical conductors, IR detectors, coatings, and many more [11,12]. Nickel sulphide (NiS) has a phase structure that is more intricate than the lanthanide sulphides [13]. NiS has distinct properties, with similarities in its two crystalline formulas and formation but differences in molecular packing [12]. At lower temperatures, rhombohedral phase (millerite) NiS was observed, while NiAs-type “symmetry” was shown at higher temperatures. The rhombohedral structure has a tetragonal pyramidal coordination of Ni atoms surrounded by five sulphur atoms, whereas NiAs displays an octahedral structure of Ni atoms coordinated with every sulphur atom. Furthermore, rhombohedral NiS exhibits temperature-independent paramagnetism-sami-metallic behaviour, whereas hexagonal NiS exhibits an antiferromagnetic semiconducting metallic phase [12]. NiS is a p-type semiconductor material with a 0.5 eV band gap and has been applied in applications such as solar cells, sensors, IR detectors, photoelectrochemical catalysts, and lithium batteries [12,13,14,15,16,17].

These attractions are sorely linked to their size, phase, surface properties, morphology, and crystal structure through the choice of preparation techniques adopted. Various approaches have been used to fabricate NiS, such as solution-processing, hydrothermal, laser ablation, and successive ionic-layer adsorption reaction methods [12,17,18,19,20]. The single-source precursors technique (SSPs) is cheap, safe, and stable with the use of one precursor. This is usually achieved through metal dithiocarbamate complexes as molecular precursors, which decompose by heating to produce QD metal sulphide materials, as reported by many research groups [21,22,23]. Changes in the dithiocarbamate organic moiety at the N-bond can give QD metal sulphide the desired morphology, size distribution, and phases [22]. Furthermore, metal dithiocarbamate complexes have better conductivity, magnetic, structural diversity, thermal, and electrochemical properties with huge potential in industrial, analytical, and biological applications [1,24,25]. The current work focuses on the optical, morphological, thermal, and structural properties of NiS prepared from *N*,*N*-dibenzyldithiocarbamate and *N*-*p*-anisidineldithiocarbamate ligands with nickel(II) complexes.

## 2. Experimental Section

### 2.1. Materials and Methods

All chemicals were of analytical grade, received from SigmaAldrich (Johannesburg, South Africa), and used without further purification. We synthesized ammonium, nickel(II) chloride hexahydrate (NiCl^2^*6H^2^O), Tri-n-octylphosphine oxide (TOPO), oleic acid, methanol, diethyl ester, carbon disulphide, *N*,*N*-dibenzyldithiocarbamate, and *N*-*p*-anisidineldithiocarbamate by slit modification of the previously published literature [23]. **[*N,N*-dibenzldtc]**. Colour: olive green; yield: 6.88 g 66%, mp 228–230 °C. ^1^H NMR (DMSO) δ 6.8–7.1 (m, 8H-C_6_H_5_), 3.3 (s, 2H-NH), 1.3 (t, 2H-CH_2_), 2.5 (s, 1H-SH). ^13^C NMR (DMSO) δ40 (-NH_2_), 51.0 (-S-C), 129 (-8H-C_6_H_5_), 207 (-CS_2_). Selected IR (cm^−1^) 1412 *v*(C-N), 1218 *v*(C-S), 3289 *v*(N-H). UV–Vis (CH_3_OH solution, nm): 315. **[*N*-*p*-anisldtc]**. Colour: olive green; yield: 20.20 g 93.87%, mp 228–230 °C. ^1^H NMR (DMSO) δ 9.42 (m, 8H-C_6_H_5_), 3.37 (s, 2H –NH), 6.89 (s, O-C_6_H_5_), 2.5 (s, 1H-SH). ^13^C NMR (DMSO) δ40 (-NH_2_), 55.8 (-S-C), 180.7 (s, O-C_6_H_5_), 124.3 (-8H-C_6_H_5_), 206 (-CS_2_). Selected IR (cm^−1^) 1412 *v*(C-N), 1218 *v*(C-S), 3391 *v*(N-H). UV–Vis (CH_3_OH solution, nm): 327.

### 2.2. Synthesis of Complexes

#### Synthesis of Bis(*N,N*-Benzyl-*N-p*-Asnisidineldithiocarbamato)Nickel(II) (Complex **1**)

**Scheme 1:**
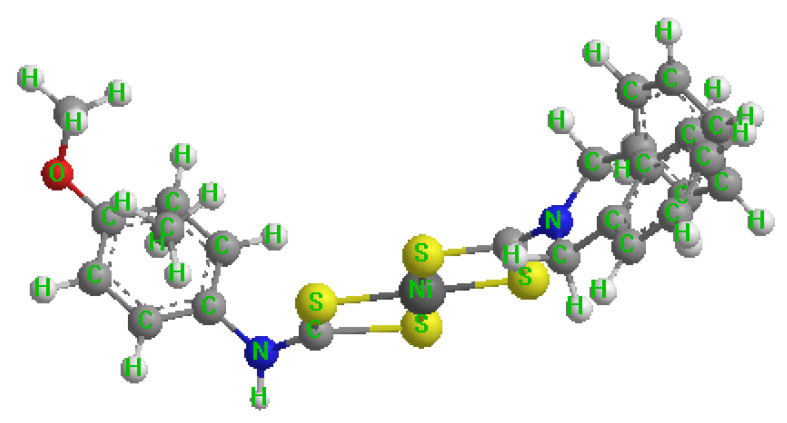
Synthesis of *bis*(*NN*-dibenzyl-*N*-*p*-anisldithiocarbamato)nickel(II) complexes.

We dissolved 2.5 mmol of *N*,*N*-dibenzyldithiocarbamate and *N*-*p*-anisidineldithiocarbamate (0.7262 g and 0.5409 g) in 15 mL of distilled water. We mixed these solutions at a ratio of 1:1:1 with (0.5939 g, 2.5 mmol) of NiCl^2^*6H^2^O dissolved in 15 mL of distilled water and stirred for 2 h at room temperature. A solid bright green precipitate was formed, washed several times with distilled water, and air dried in a calcium vacuum to yield the following end product as seen in Scheme 1: **[Ni[*N*,*N*-benz-*N*-*p*-anisldtc]**. Colour: bright green; yield: 68%, mp 232–234 °C. ^1^H NMR (DMSO) δ 7.47–9.46 (m, 8H-C_6_H_5_), 4.04–5.05 (s, 2H-NH), 6.86 (s, O-C_6_H_5_), 2.51 (s, 1H-SH). ^13^C NMR (DMSO) δ40 (-NH_2_), 51.3–55.7 (-S-C), 181.9 (s, O-C_6_H_5_), 126.5–157 (-8H-C_6_H_5_), 210 (-CS_2_). Selected IR (cm^−1^) 1428 *v*(C-N), 1140 *v*(C-S), 3215 *v*(N-H), 518 *v*(M-S). UV–Vis (CH_3_OH solution, nm): 265. We followed same path for the synthesis of *N*,*N*-dibenzyldithiocarbamate with nickel(II) complex at ratio (2:1) labelled as **[Ni[*N*,*N*-benzldtc]** and *N*-*p*-anisidineldithiocarbamate with nickel(II) complex at ratio (2:1) labelled as **[Ni[*N*-*p*-anisldtc]**. **[Ni[*N*,*N*-benzldtc]**. Colour: bright green; yield: 74%, mp 234–236 °C. ^1^H NMR (DMSO) δ 7.47–9.46 (m, 8H-C_6_H_5_) 3.7 (s, 2H –NH), 1.32 (t, 3H-CH_2_), 3.30 (s, 1H-SH). ^13^C NMR (DMSO) δ 126–143 (-C_6_H_5_), 40 (-CH_2_), 203 (-CS_2_), 52 (-C–NH). Selected IR (cm^−1^) 1495 *v*(C-N), 1228 *v*(C-S), 3028 *v*(N-H), 518 *v*(M-S). UV–Vis (CH_3_OH solution, nm): 259. **[Ni[*N*-*p*-anisldtc]**. Colour: green; yield: 78%, mp 234–236 °C. ^1^H NMR (DMSO) δ 6.82–9.43 (m, 8H-C_6_H_5_), 3.6 (s, 2H –NH), 3.32 (t, 3H-CH_2_), 2.5 (s, 1H-SH). ^13^C NMR (DMSO) δ 137–146 (-C_6_H_5_)40 (-CH_2_), 206 (-CS_2_), 55.9 (-C–NH). Selected IR (cm^−1^) 1467 *v*(C-N), 1030 *v*(C-S), 3166 *v*(N-H), 520 *v*(M-S). UV–Vis (CH_3_OH solution, nm): 277. 

### 2.3. Synthesis of Nickel Sulfide Nanoparticles

In a neck-bottom flask, we dissolved 0.2 g of bis(*N,N*-dibenzyl-*N-p*-anisidineldithiocarbamato) nickel(II) as Ni[*N,N*-benz-*N-p*-anisidineldtc] [2] in 4 mL of oleic acid mixed with 3 g of hot coordinating solvent TOPO. We observed the 20–30 °C initial heating of the TOPO in the bottom flask for about 20 min. We heated the reaction to 260 degrees Celsius and held there for one hour. We took the dark aqueous solution from the flask at a reduced temperature of around 70 °C. We added an additional 50 mL of methanol to remove the excess capping agent and toxic solvent by centrifugation at 2000 rpm for 30 min, followed by drying with an air vacuum. We labelled the prepared NiS as NiSa, while we used Ni[*N,N*-benzldtc] and Ni[*N-p*-anisldtc] to fabricate NiSb and NiSc under similar conditions and procedures.

### 2.4. Characterization

We elevated the optical properties, surface morphology, thermal stability, elemental compositions, and thickness of the NiS nanoparticles through the following techniques: field emission scanning electron microscope (FE-SEM, S-4200, Hitachi, Munich, Germany) coupled with energy dispersive X-ray spectroscopy (EDS) on-system, operating at a voltage of 15 kV. We identified the size distributions of the three samples using JEOL JEM 2100 (Pleasanton, CA, USA) Transmission Electron Microscope (TEM) operating at 200 kV. We established their structural patterns using X-ray diffraction (XRD) analysis by Cu Ka radiation run at 40 mA and 40 kV. We obtained surface roughness using an atomic force microscope (JPK NanoWizard II AFM, JPK Instruments, Berlin, Germany) at a scan rate of 0.8 Hz. We carried out TGA analysis at temperatures ranging from 30 to 600 degrees Celsius at a rate of 10 degrees Celsius per min^−1^. We achieved Fourier transform infrared spectroscopy (FTIR) analysis through the aid of Bruker Platinum ATR Model Alpha (Waltham, MA, USA). We used the PerkinElmer instrument of the model LAMBDA 365 and LS 45 fluorimeters to understand the optical properties (UV-Vis and PL analysis) of the three samples. We carried out NMR analysis using a Bruker AV-400 spectrometer (Waltham, MA, USA) working at 400.13 MHz, 300 K, and a spinning rate of 4 kHz.

## 3. Results and Discussion

### 3.1. TGA and FTIR Analysis

**Figure 1:**
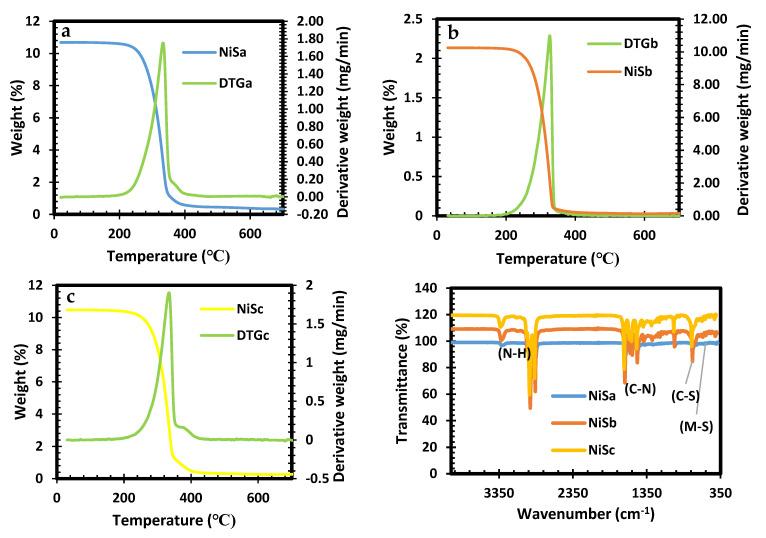
TGA (**a**–**c**) of [Ni[*N*,*N*-benz-*N*-*p*-anisldtc], [Ni[*N*,*N*-benzldtc], and [Ni[*N*-*p*-anisldtc] complexes as NiSa, NiSb, and NiSc and FTIR spectras (**d**) of NiSa, NiSb, and NiSc nanoparticles.

The thermal decomposition of Ni[*N*,*N*-benz-*N*-*p*-anisldtc], Ni[*N*,*N*-benzldtc], and Ni[*N*-*p*-anisldtc] is shown in Figure 1a–c. The onset decomposition heat for Ni[*N*,*N*-benz-*N*-*p*-anisldtc] and Ni[*N*-*p*-anisldtc] complexes is 275 °C, while the final decomposition heat is 333–334 °C, resulting in NiSa and NiSc conversion. Ni[*N*,*N*-benzldtc] has a decomposition profile onset at 270 °C and a single-step decomposition at 348 °C for the formation of NiSb. The three samples revealed a complete loss in their final mass residual. This occurrence is consistent with the reports by Ref. [26] on the thermolysis of Ni complexes. An FTIR spectra analysis was taken (see Figure 1d), which affirmed the presence of typical constituents of dithiocarbamates and Ni-S compounds. The absorption bands of N-H at 3324 cm^−1^ and the vibration modes of C-S and C-N bands at 972–729 cm^−1^ and 1479–1643 cm^−1^ revealed the interface between the aromatic precursors and metal ion substances. The presence of the vibration frequencies at 587 and 423 cm^−1^ correlates to the metal with sulphur atoms M-S coordination, which agrees with the report in Ref. [27].

### 3.2. XRD

**Figure 2:**
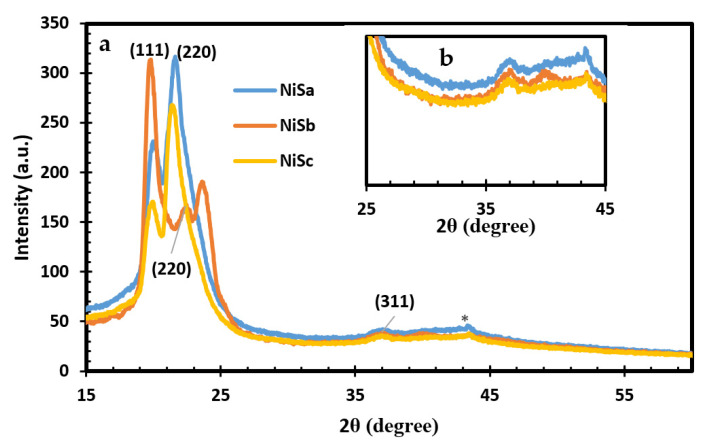
XRD spectra for NiSa, NiSb, and NiSc as (**a**) and inset Figure as (**b**) nanoparticles.

Figure 2a and the inset Figure 2b show the patterns with the presence of one-phase nickel sulphide nanoparticles. The diffraction peaks for NiSa and NiSc at 19.82°, 21.68°, and 37.02° and for NiSb at 19.82°, 22.52°, and 37.02° exhibited by the three samples correspond to the (111), (220), and (311) planes of cubic NiS nanoparticles (ICDD card number 043-1469). The XRD pattern illustrates the polycrystalline nature of the samples, with some impurities from the coordinating solvent around 19° to 24° being observed. The small peak observed at 43.64° is ascribed to pure nickel, which is similar to the observation by Ref. [27] for cubic NiS nanoparticles. The results of this study on the formation of NiS with a dithiocarbamate complex via thermolysis at high temperatures are consistent with previous research [28,29].

### 3.3. UV-Vis 

**Figure 3:**
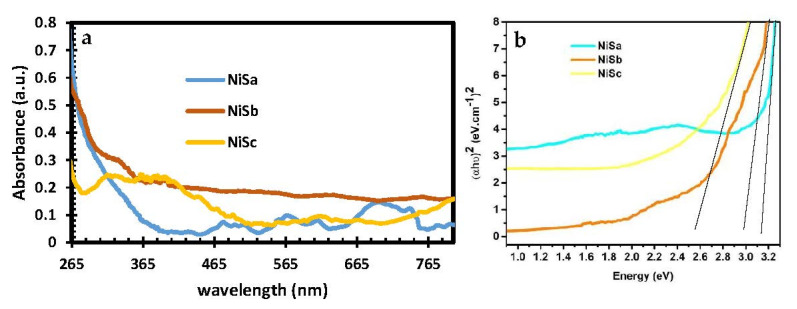
UV—Vis (**a**) and Tauc plot (**b**) spectra for NiSa, NiSb, and NiSc nanoparticles.

The optical spectra along with the estimated Tauc plots for the NiSa, NiSb, and NiSc nanoparticles synthesized from mixed and single molecular precursors with TOPO coordinating solvent are shown in Figure 3a,b. The optical spectra for NiSa and NiSc nanoparticles have an absorbance response in the blue-shifted regions at 482 nm and 446 nm. The obtained optical properties for both nanoparticles have better size distributions as a result of the quantum confinement effect, which is better than the previous studies using HDA capping agents [30]. The absorption properties of NiSb show a lower response wavelength at 388 nm, 629 nm, and 758 nm. The optical characteristics from this study suggest that the prepared nanoparticles have strong potential for solar cell applications due to their absorption in the blue shift regions. By extrapolating the linear part of the data in the Tauc plots, optical band gap values of 3.12 eV, 2.95 eV, and 2.5 eV were obtained for NiSa, NiSb, and NiSc, respectively.

### 3.4. PL Analysis

**Figure 4:**
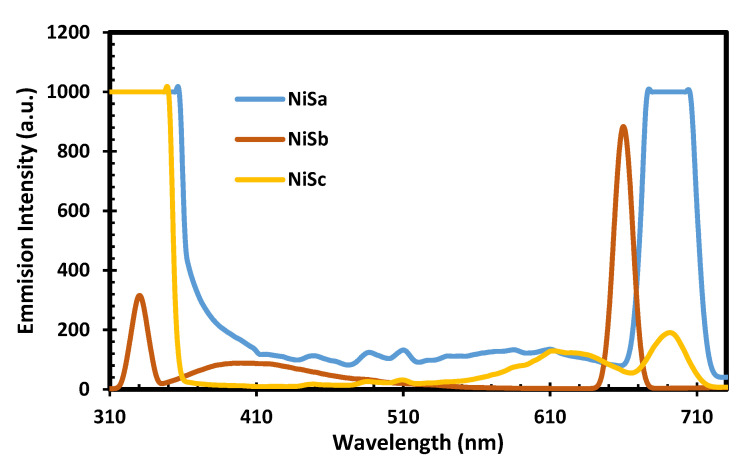
PL spectra for NiSa, NiSb, and NiSc nanoparticles.

The PL spectra of NiSa, NiSb, and NiSc nanoparticles with an excitation wavelength of 350 nm are seen in Figure 4. The emission peaks at 447 nm and 488 nm for NiSa and NiSc are due to defects. The corresponding emission peaks at 625 nm and 684 nm for NiSc, and 612 nm and 662 nm for NiSb, are attributed to the red shift. These emission peaks are a strong indication of a larger size distribution or anisotropic particles. The observed emission in this study is similar to that in the literature [27,31]. 

### 3.5. TEM

**Figure 5:**
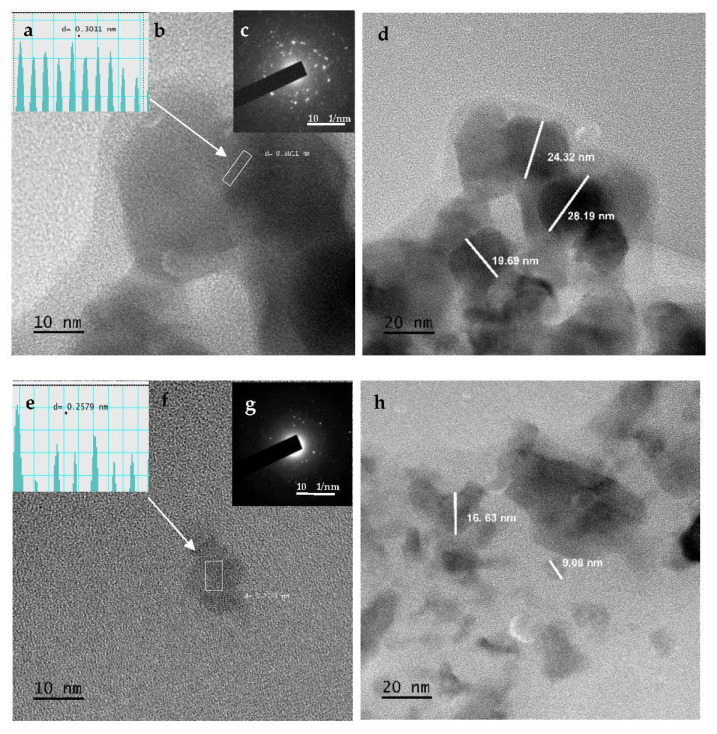

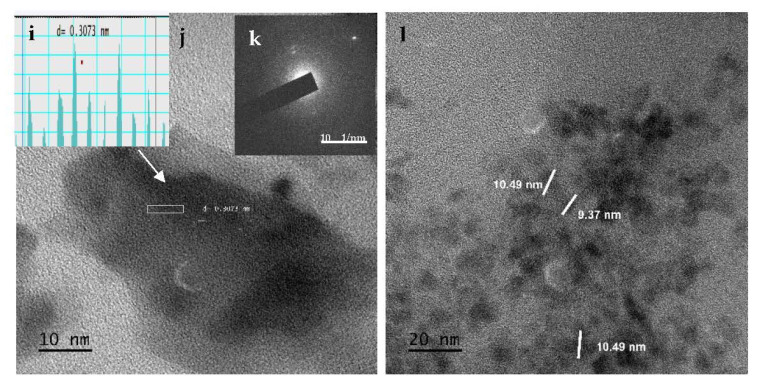
TEM images, d-spacing, and SEAD for NiSa (**a**–**d**), NiSb (**e**–**h**), and NiSc (**i**–**l**) nanoparticles.

The TEM technique was used to obtain a detailed analysis of the particle structure. The size distribution of the three nanoparticles was found to be between 19.69 and 28.19 nm for NISa, 9.08 and 16.63 nm for NISb, and 9.37 and 10.49 nm for NISc. The TEM image further verifies the uniformity of NiSa nanoparticles with a large number of mesopores and less aggregation, as seen in Figure 5b,d. The lattice d-spacing of 0.30 nm is in correlation with the (111) plane of cubic NiS [32], as seen in the inserted Figure 5b, which is in agreement with the report by Ref. [33]. The porous and loose structure of NiSa has been shown to improve electrocatalytic activity by increasing electrolyte contact [33]. The compact nature of NiSb agglomerates was demonstrated in Figure 5f,h. Figure 5f,h reveal the spherical structure of NiSb nanoparticles through the aid of TOPO coordinating solvent surface passivation, leading to the fabrication of nanopores. The lattice spacing of 0.25 nm corresponds to the (111) plane of NiS (as shown in the inserted Figure 5g), which is in good agreement with the literature [34]. TEM images of NISc display spherically shaped particles (see Figure 5j,l and the inset in Figure 5i,k) due to coalescence, which involves the interaction between the particle–particle with several neighbouring nanospheres. This phenomenon is associated with the reduction of surface energy in nanoparticles as a result of their small dimensions, which results in a high reactive surface energy and unstable nanoparticles [30,35,36]. The lattice d-spacing of 0.30 nm correlates to the plane (111) of cubic NiS nanoparticles, while the SAED image reveals bright spots and the crystallinity nature of NiSc nanoparticles. This study further affirmed the report by Ref. [30] on the role of long alkyl chains in changing the shape and thermal stability of nanoparticles. The summary of the three samples prepared by the SSPs method is found in Table 1.

### 3.6. AFM

**Figure 6:**
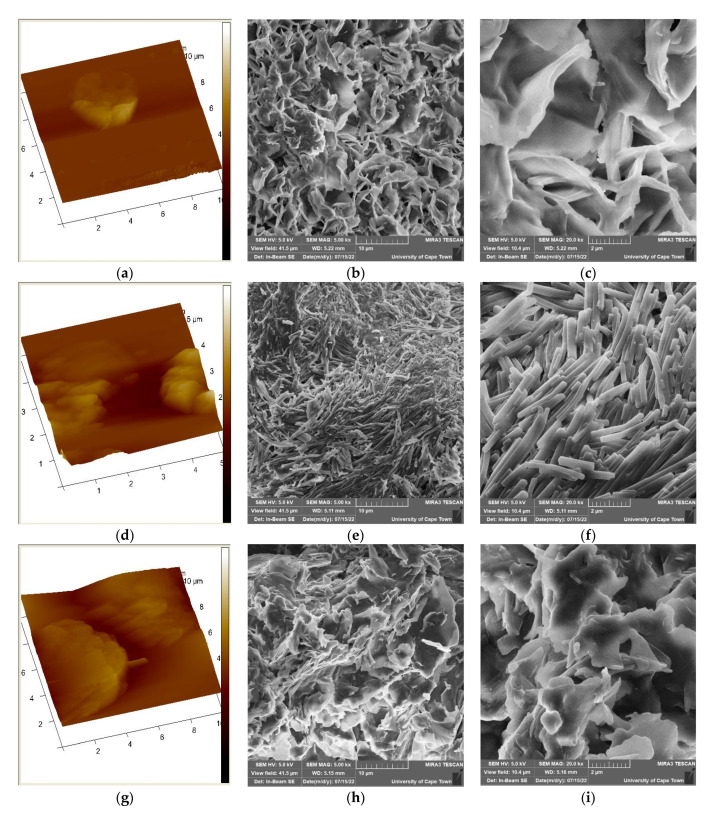
AFM (**a**,**d**,**g**) and SEM images for the prepared NiSa (**b**,**c**), NiSb (**e**,**f**), and NiSc (**h**,**i**) nanoparticles.

The surface roughness of NiSa, NiSb, and NiSc nanoparticles was studied using AFM, as shown in Figure 6a,d,g. The quantitative details of the surface profile, such as average roughness (Ra), 3D dimensional simulation, and root mean square roughness (Rq), were obtained from the samples. The topographical view confirms that three metal sulphides are characterized by indentations and irregular surfaces. The Rq was found at 93.8, 75.7, and 425 nm for NiSa, NiSb, and NiSc, respectively, while the Ra for NiSa, NiSb, and NiSc was found at 46.8, 49.1, and 334 nm, respectively. The slight difference in surface roughness corresponds to the relative changes in particle size. The particle size can influence the electron transfer speed and current density [37,38]. The AFM measurements correspond with results obtained from the TEM.

### 3.7. FE-SEM

Figure 6b,c,e,f,h,i illustrate the morphological evolution of the as-prepared NiS for the three samples. The images in Figure 6b,c,e,f,h,i reveal a clearly distinguishable flower/star-like morphology for NiSa and NiSc with some clustered nanoparticles. The formation of an irregular flower/star finer morphology for NiSa and NiSc is linked to their larger surface area, which promotes higher reducibility, better size distribution, and superior catalytic reactions [39,40]. On the other hand, NiSb reveals compact nanorod-shaped nanoparticles, as seen in Figure 6e,f, which supports the TEM results. The predominant nanorods observed in NiSb are vital aspects of materials that can enhance electrolyte transport, photoactivity, and the optimization of light absorbance. To the best of our knowledge, very few studies on the flower/star-like and nanorod shapes have been reported, and none with dithiocarbamate as a molecular precursor for NiS nanoparticles [41,42,43].

### 3.8. EDS

**Figure 7:**
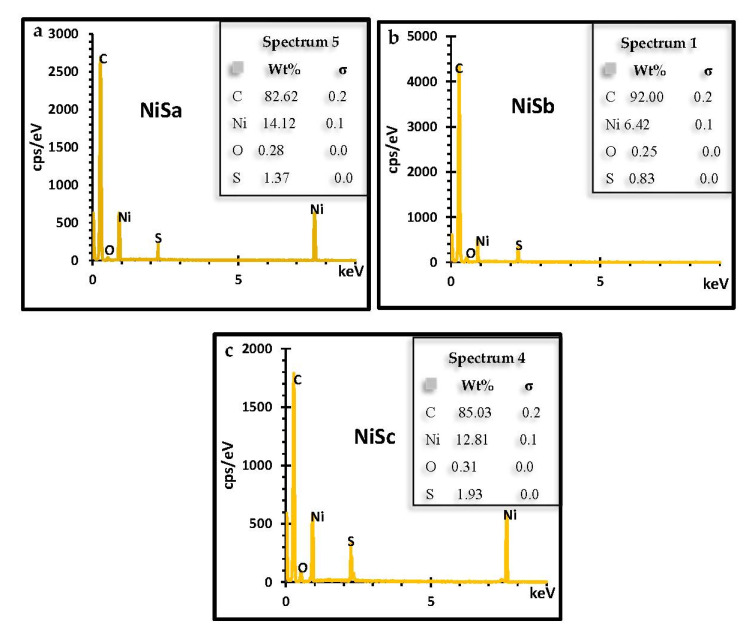
EDS spectra of NiSa (**a**), NiSb (**b**), and NiSc (**c**) nanoparticles.

Figure 7a–c confirm the elemental composition and purity of the synthesized NiS nanoparticles through EDS. The annealed nanoparticles reveal the presence of strong Ni and S in the three samples. The organic elements of Ni and S observed from the profiles of the three samples originated from the molecular precursors, which indicates the successful synthesis of NiS and affirms the FTIR results [44].

## 4. Conclusions

In conclusion, the SSP route is a risk-free, low-cost, and simple method of synthesizing quantum dot nickel sulphide using a molecular precursor. The TOPO-capped agent with the mixed and single precursor influences the novel flower/star-like and nanorod nickel sulphide NiS quantum dot nanostructures. This has a significant impact on particle sizes, surface roughness, morphologies, and optical properties. Surface roughness and size distribution can influence the electron transfer speed and the current density for solar cell applications. The emission peaks in the blue and red shift regions are strong indications that these materials can absorb the entire solar spectrum, giving rise to better efficiency in solar cell applications, such as quantum dot-sensitized solar cells. Moreover, flower/star-like and nanorod materials are vital aspects that can enhance electrolyte transport, photoactivity, and light-absorbance optimization.

## Figures and Tables

**Table 1:** Summary of NiSa, NiSb, and NiSc nanoparticles prepared by SSPs approach.

Samples	Particle Size (TEM Images (nm))	*d*-Spacing (nm)	PL Emission (nm)	Band Gap from Taucs Plot (eV)	Surface Roughness from AFM (nm)	Atomic Percentage from EDS	
						Ni	S
NiSa	19.69–24.32	0.30	447	3.12	46.8	14.12	1.37
NiSb	9.08–16.63	0.25	662	2.95	49.1	6.42	0.83
NiSc	9.37–10.49	0.30	488	2.5	334	12.81	1.93

## Data Availability

Not applicable.
